# Supplementary material for: Spidroin N-terminal domain forms amyloid-like fibril based hydrogels and provides a protein immobilization platform
Source: Nat Commun. 2022 Aug 15;13:4695. doi: 10.1038/s41467-022-32093-7 (PMC9378615; doi:10.1038/s41467-022-32093-7)
Supplement: Supplementary file 1 — Supplementary information [file 41467_2022_32093_MOESM1_ESM.pdf]

Supplementary Information

Spidroin N-terminal domain forms amyloid-like fibril based hydrogels and provides a protein immobilization platform

Tina Arndt[1], Kristaps Jaudzems[2], Olga Shilkova[1], Juanita Francis[1], Mathias Johansson[3], Peter R Laity[4], Cagla Sahin[5] , Urmimala Chatterjee[1], Nina Kronqvist[1], Edgar Barajas-Ledesma[5], Rakesh Kumar[1], Gefei Chen[1], Roger Strömberg[1], Axel Abelein[1], Maud Langton[3], Michael Landreh[5], Andreas Barth[6], Chris Holland[4], Jan Johansson[1], Anna Rising[1,7,*]

[1]Department of Biosciences and Nutrition, Karolinska Institutet, Neo, Blickagången 16, Huddinge 141 52, Sweden.

[2]Department of Physical Organic Chemistry, Latvian Institute of Organic Synthesis, Riga, LV-1006, Latvia.

[3]Department of Molecular Sciences, Swedish University of Agricultural Sciences, Uppsala 750 07, Sweden.

[4]Department of Materials Science and Engineering, The University of Sheffield, Sir Robert Hadfield Building, Mappin Street, Sheffield S1 3JD, United Kingdom.

[5]Department of Microbiology, Tumor and Cell Biology, Karolinska Institutet, Solnavägen 9, 171 65 Solna, Sweden.

[6]Department of Biochemistry and Biophysics, The Arrhenius Laboratories for Natural Sciences, Stockholm University, 10691 Stockholm, Sweden.

[7]Department of Anatomy, Physiology and Biochemistry, Swedish University of Agricultural Sciences, Uppsala 750 07, Sweden.

* Corresponding author (email: anna.rising@ki.se)

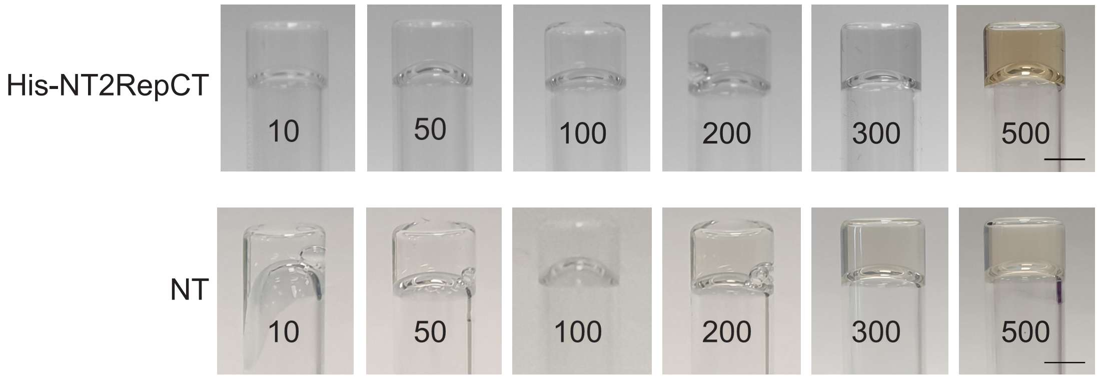

Supplementary Fig. 1: Vial inversion tests of His-NT2RepCT and NT hydrogels. Photographs of NT2RepCT hydrogels (top row) and NT hydrogels (bottom row) with a concentration of 10, 50, 100, 200, 300 and 500 mg/ml formed at 37 °C (scale bars are 5 mm).

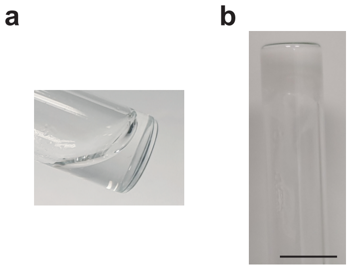

Supplementary Fig. 2: Vial inversion tests. a Native spider silk dope (50 mg/ml) in 20 mM Tris-HCl buffer after 21 days at 37 °C. **b** NT (100 mg/ml) with 154 mM NaF after incubation at 37°C (scale bar is 5 mm).

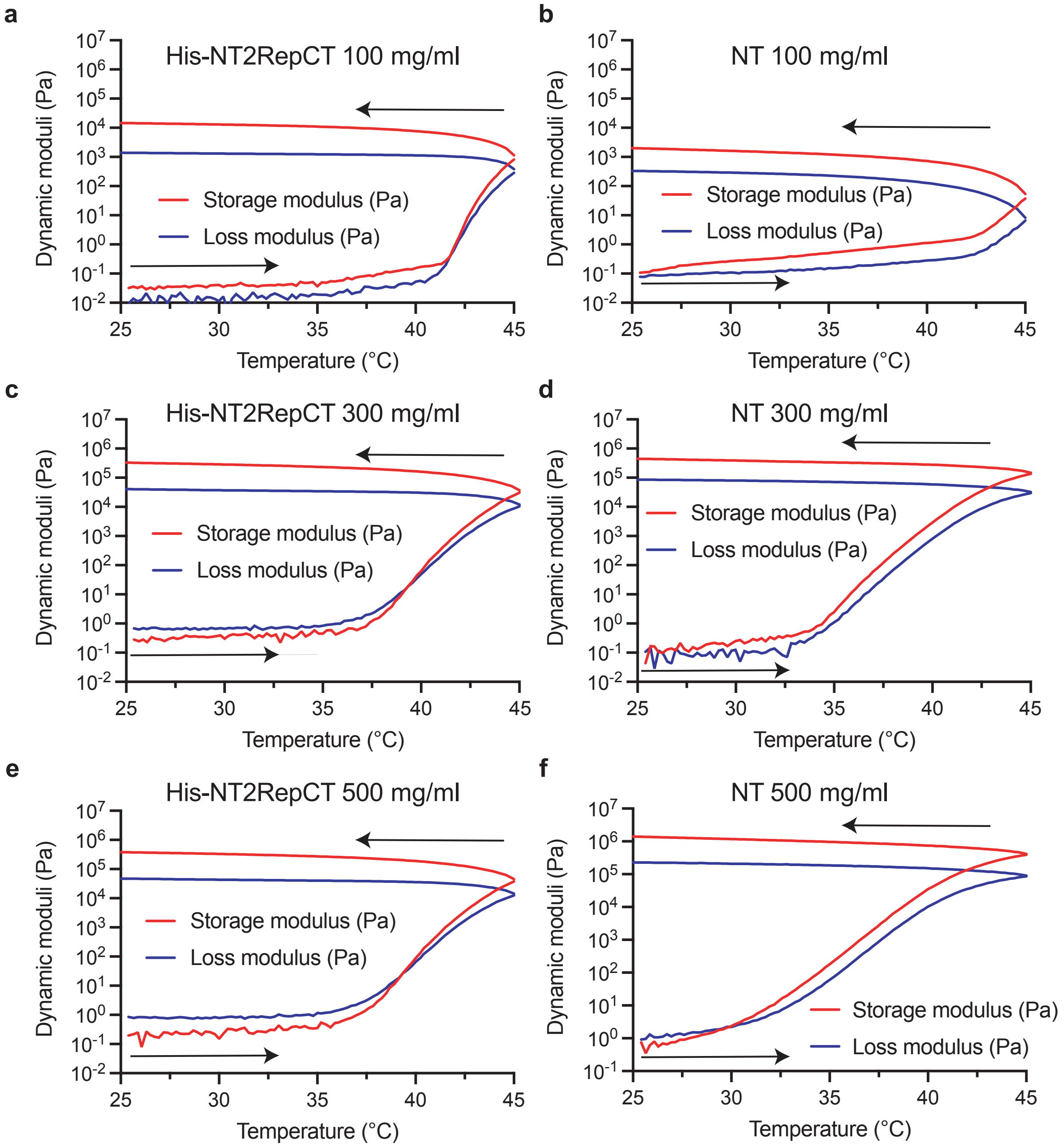

Supplementary Fig. 3: Temperature ramp during oscillatory measurements on His-NT2RepCT and NT solutions with concentrations of **a, b** 100 mg/ml **c, d** 300 mg/ml, **e,f** 500 mg/ml. Red, G′ storage modulus; blue, G″ loss modulus.

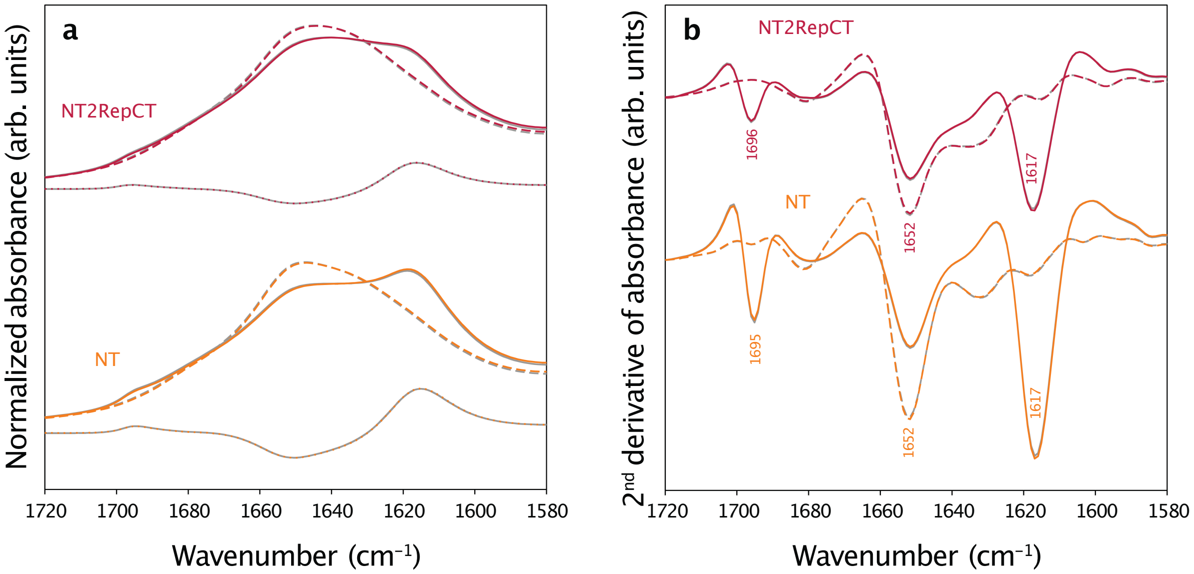

Supplementary Fig. 4: FTIR spectra and difference spectra of His-NT2RepCT and NT with and without correction for the wavenumber dependency of the penetration depth in ATR-FTIR spectroscopy. a Area-normalized absorbance spectra of His-NT2RpeCT and NT (both at 500 mg/ml) before (dashed lines) and after (full lines) incubation at 37 °C. The original spectra are shown in color and correspond to those shown in Fig. 3. They were obtained from the negative logarithm of the intensity ratios with and without sample. The corrected spectra are shown in gray. The correction has very little impact in the limited wavenumber range of interest. Each dotted colored line is the difference of a spectrum after gelation minus a spectrum before gelation. The underlying gray full-line spectra are the respective differences of the corrected spectra. The difference spectra reveal a gain in β-sheet structure at 1695 and 1617 cm^{-1} and a loss in α-helix structure at 1651 cm^{-1} as well as a possible loss of a β-sheet component near 1635 cm^{-1}. These structural differences are larger for NT than for His-NT2RepCT. **b** Second derivatives of area-normalized absorbance spectra of His-NT2RpeCT and NT (both at 500 mg/ml) before (dashed lines) and after (full lines) incubation at 37 °C. Colored lines are calculated from the original spectra and gray lines from the corrected spectra. Again, the effects of the correction are hardly visible. The component bands of the amide I band show up as negative bands in second derivative spectra where they can be better distinguished than in absorbance spectra. The second derivative spectra clearly reveal the appearance of β-sheet bands at 1695 and 1617 cm^{-1} upon gelation.

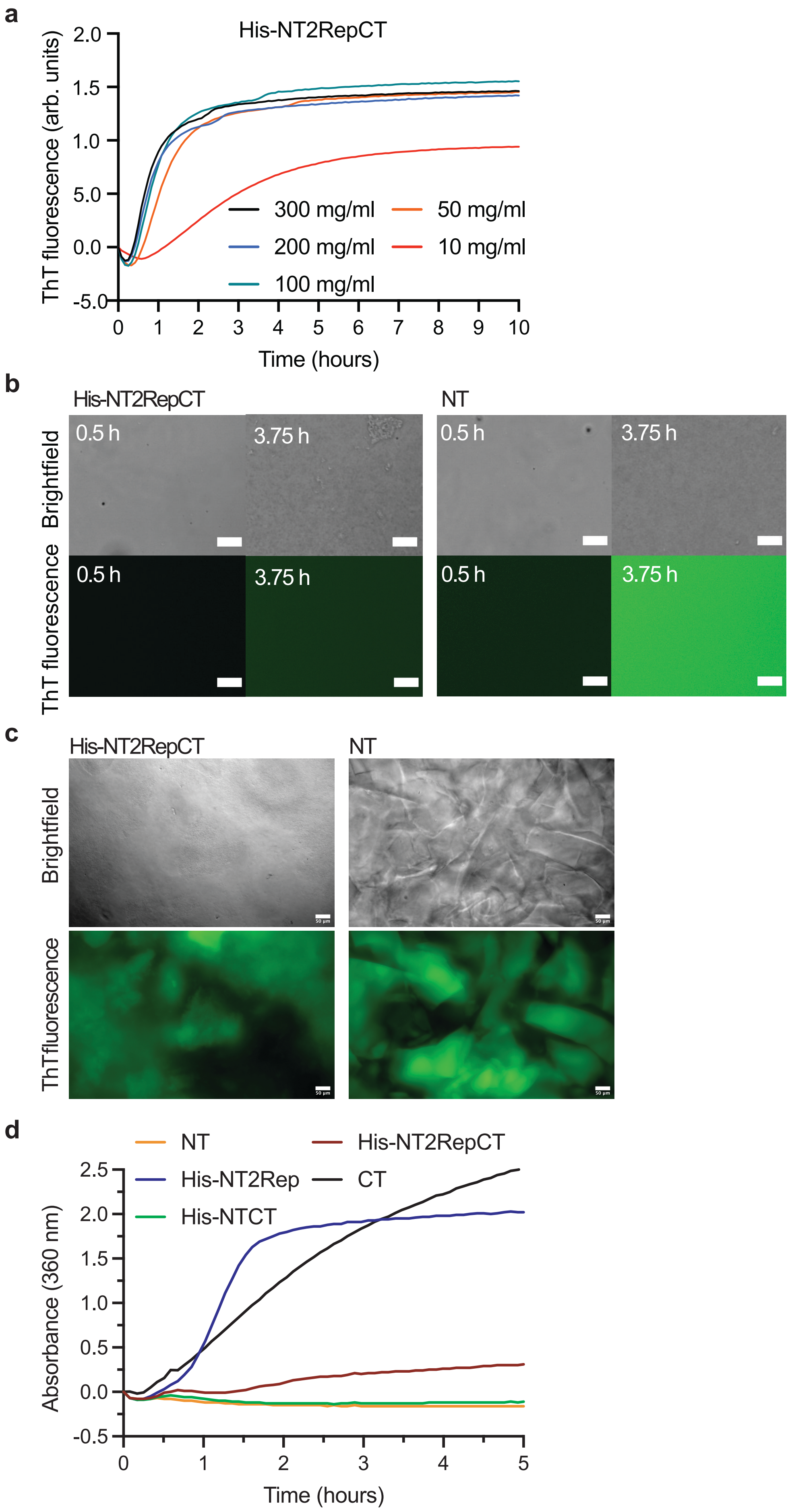
a
His-NT2RepCT
ThT fluorescence (arb. units)
2.0
1.5
1.0
5.0
0.0
-5.0
0
1
2
3
4
5
6
7
8
9
10
Time (hours)
300 mg/ml
50 mg/ml
200 mg/ml
10 mg/ml
100 mg/ml
b
His-NT2RepCT
NT
Brightfield
ThT fluorescence
0.5 h
3.75 h
0.5 h
3.75 h
0.5 h
3.75 h
0.5 h
3.75 h
c
His-NT2RepCT
NT
Brightfield
ThTfluorescence
d
NT
His-NT2RepCT
His-NT2Rep
CT
His-NTCT
Absorbance (360 nm)
2.5
2.0
1.5
1.0
0.5
0.0
-0.5
0
1
2
3
4
5
Time (hours)

Supplementary Fig. 5: Binding of ThT to His-NT2RepCT and NT and turbidity of hydrogels. a ThT fluorescence of His-NT2RepCT at different concentrations at 37 °C without shaking. **b** Brightfield (top row) and fluorescence microscopy (bottom row) of His-NT2RepCT and NT with 5 μM ThT at 37°C at 30 min and 3.75 hours incubation. Scale bars are 25 μm. Experiments were repeated twice with similar results. **c** Brightfield and fluorescence microscopy of NT and His-NT2RepCT gels that were formed in the presence of ThT, then broken to pieces in Tris-HCl buffer containing 5 μM ThT. Both types of gels fluoresce, which means they have bound the β--sheet specific Thioflavin T (ThT), while the ThT containing buffer is black. Scale bars are 50 μm. Experiments were repeated twice with similar results. **d** Turbidity measurements over time of the different recombinant spidroins at concentrations of 100 mg/ml at 37 °C.

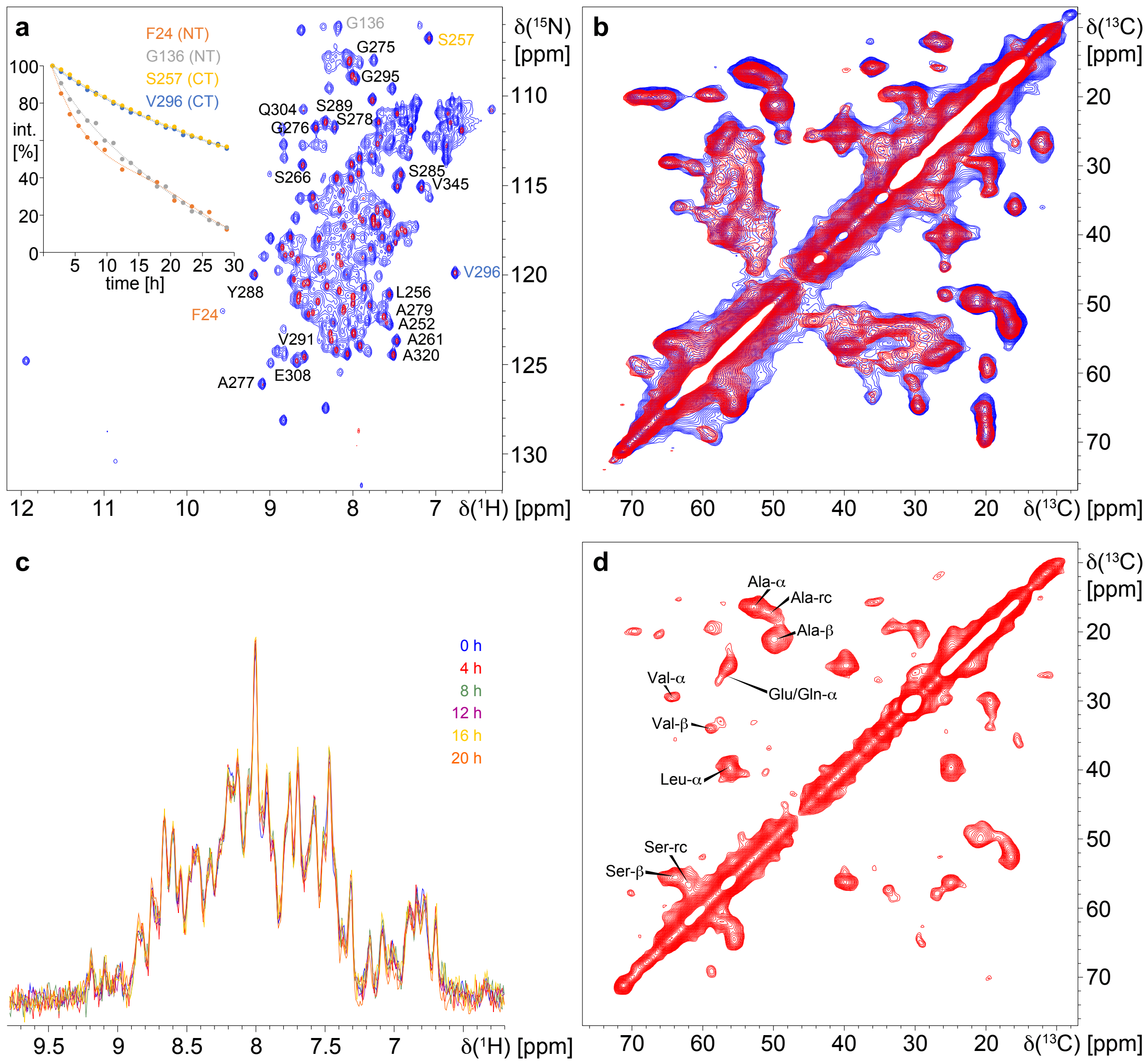

e

GAVNRLSSAEAASRVSSNIAAIASGGASALPSVISNIYSGVVASGVS[SNEALIQALLELLSALVHVLSSA]SIG

NVSSVGVDSTLNVVQDSVGQYVG

Supplementary Fig. 6: NMR spectroscopy of His-NTCT and CT. a 2D ^{15}N-HSQC spectra of a 10 mg/ml His-NTCT solution before (blue) and after (red) incubation at 37 °C for 48 h. Isolated cross-peaks in the red spectrum and F24, G136 in the blue spectrum are assigned using one-letter amino acid symbols and residue numbers. The inset shows signal intensity versus time for selected residues from the NT and CT domains. **b** Overlay of MAS solid-state DARR spectra of His-NTCT (red) and His-NT2RepCT (blue) hydrogels **c** 1D ^{15}N-HSQC spectra of a 10 mg/ml CT solution during incubation at 37 °C for 20 h. **d** Solid-state RFDR spectra of CT hydrogel. Cα/Cβ correlations of residues observed in the RFDR spectrum and their secondary structure determined by comparison with the chemical shifts of model peptides and values obtained from statistical data[1,2] are

indicated. **e** Sequence of CT with Ala (in α- and β-secondary structure) and Leu residues (mainly in α-secondary structure) indicated in green and in yellow, respectively. The location of helix 4 in the center of CT dimer interface, which contains most leucines, is shown in square brackets.

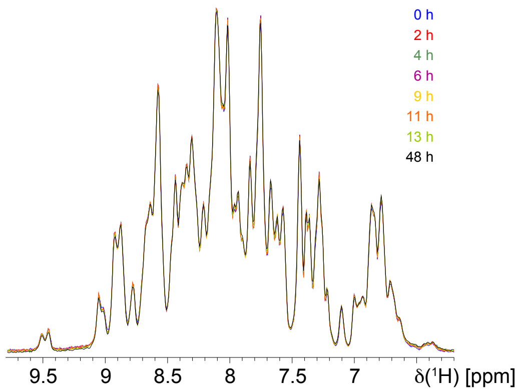

Supplementary Fig. 7: 1D ^{15}N-HSQC spectra of NT*(10 mg/ml) over the course of incubation at 37 °C for 48 h.

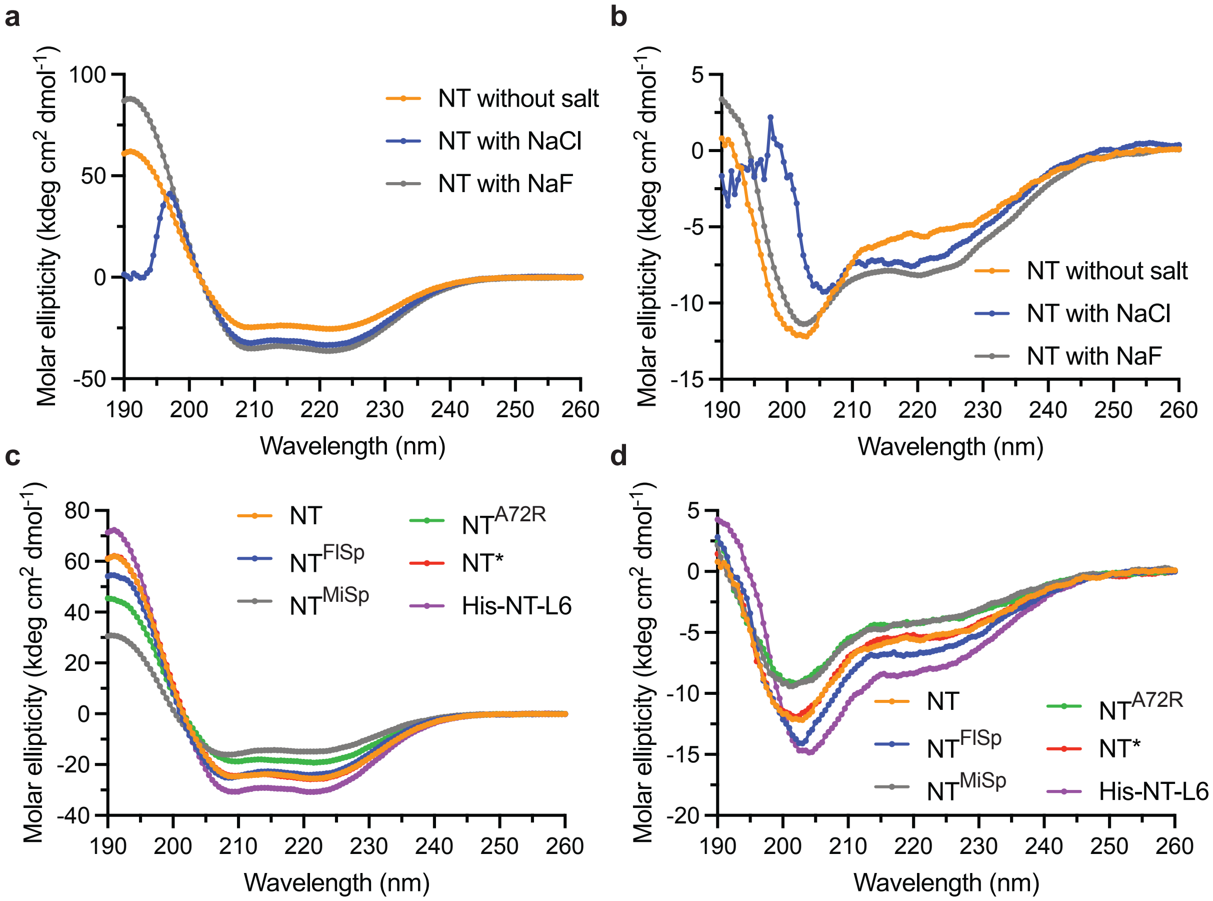

Supplementary Fig. 8: CD spectra of NTs. a CD spectra of NT without salt and with NaCl and NaF, respectively, at 25°C and **b** at 95°C. **c** CD spectra of NT and different NT mutants at 25°C (NT and NT* spectra overlap) and **d** CD spectra of NT and different NT mutants at 95°C.

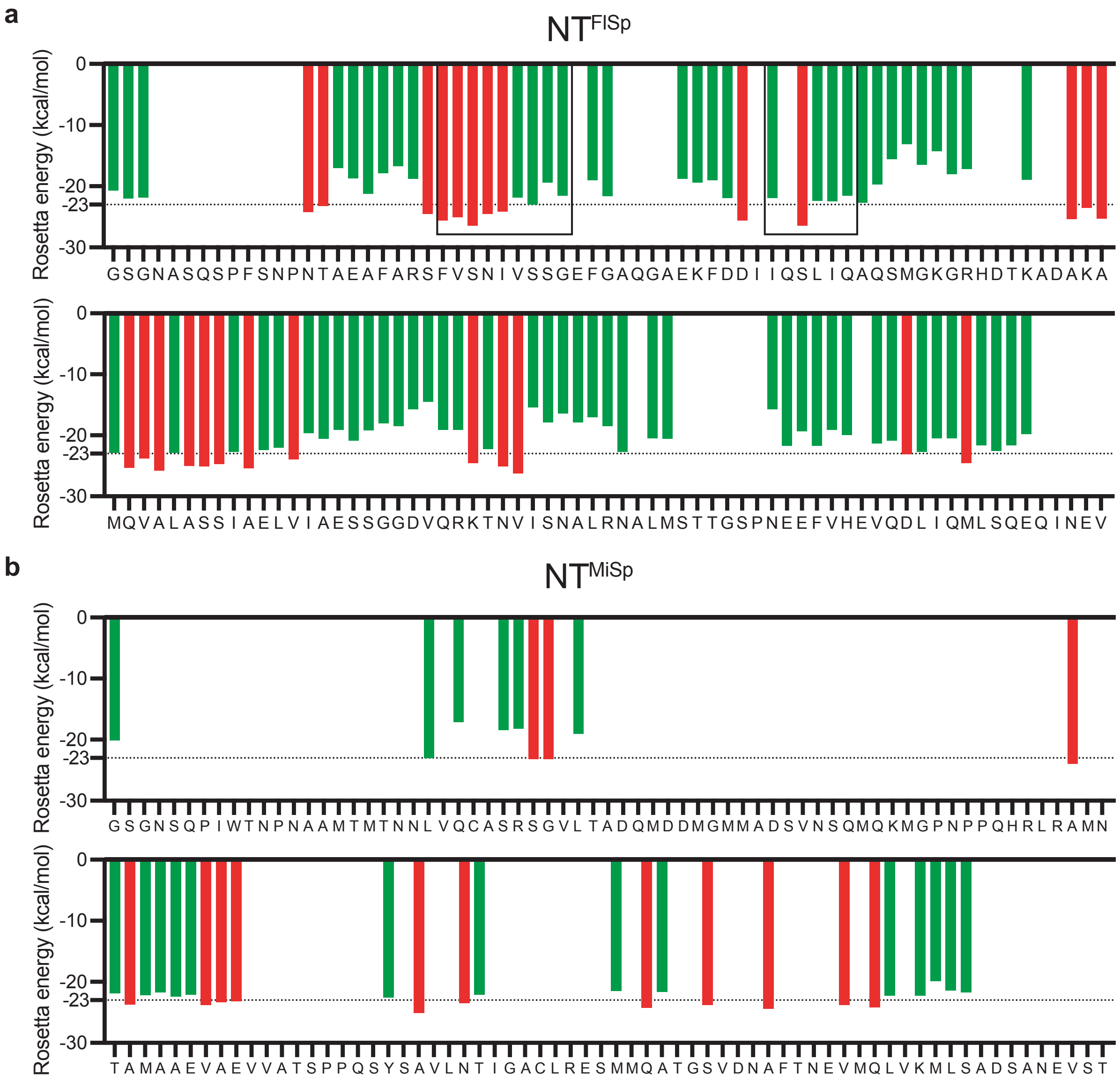

Supplementary Fig. 9: Fibrillation propensity profile of a NT^{FlSp} and b NT^{MiSp} [3], (https://services.mbi.ucla.edu/zipperdb/). Bars show Rosetta energies, red bars indicate hexapeptides with high fibrillation propensities (Rosetta energies below -23 kcal/mol; below dotted line). Green bars indicate Rosetta energies above the threshold which are segments that are unlikely to form fibrils. Squares indicate a predicted amyloidogenic regions with the Waltz algorithm 52, (https://waltz.switchlab.org/).

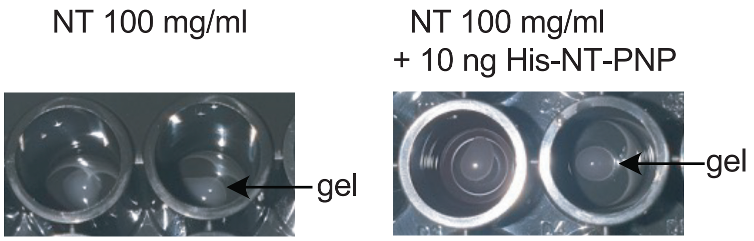

Supplementary Fig. 10: Hydrogels for PNP activity assays. Photographs of intact NT (100 mg/ml) and NT (100 mg/ml) + His-NT-PNP (10 ng) hydrogels after the assay.

Supplementary Table 1: Overview of spidroin hydrogels, their method of gelation, gelation time, concentrations, elastic modulus, and transparencies. N.s. – not stated. * not stated how gelation time was determined. Gelation time determined by increase in turbidity**, vial inversion***, rheology ****

Reference	Spidroin name	Method of gelation	Gelation time	Concentration (mg/ml)	Elastic modulus (kPa)	Transparency
Rammensee et al., 2006[5]	ADF-4(C16)	APS, Rubpy	days – week*	5-30	≈ 1	n.s.
Schacht et al., 2011[6]	eADF4(C16)	APS	80 hours**	30-70	110	opaque
Schacht et al., 2015[7]	eADF4(C16)	37 °C	O.N.*	30	0.2	opaque
Qian et al., 2015[8]	NcCT, AdCT, EaCT and MaSpI4C	2 °C/ 65 °C	10 min ***	150	0.5/ 0.001	transparent at 2°C
DeSimone et al., 2016[9]	eADF4(C16)	ionic (DMEM)	10-15 hours**	30	n.s.	opaque
Thamm et al., 2017[10]	eMaSp1s	37 °C	60 h**	30, 50, 70	5 - 18	opaque
DeSimone et al., 2017[11]	eADF4(C16),	37 °C	O.N.**	30-70	5	opaque

Humenik et al., 2020[12]	eADF4(C16)	100 mM KPi	24 h*	n.s.	n.s.	n.s.
Song et al., 2021[13]	E16C	22 °C/ 57 °C	n.s.	150	1/ 0.001	transparent
		37 °C	40 min ****	150	0.01	n.s.
Neubauer et al., 2021[14]	eADF4(C16), eADF4(κ16) and eADF(Ω16)	DMSO	n.s.*	10-30	0.1 kPa	transparent
Present study	NT2RepCT	37 °C	1 hour ***	100	14.6	transparent
			30 min ***	300	50.7	
			25 min ***	500	330.9	
	NT		30 min ***	100	2.0	
			10 min ***	300	439.5	
			10 min ***	500	1369.3	

Supplementary Table 2: Methionine content, grand average of hydropathy [15], (https://web.expasy.org/protparam/), the percentage of Rosetta energies below -23 kcal/mol, number of amyloidogenic regions as predicted by Waltz [4], (https://waltz.switchlab.org/)of NT^{MaSp}, NT^{FlSp} and NT^{MiSp} constructs.

	NT^{MaSp}	NT^{FlSP}	NT^{MiSp}
Methionine content	7.4 %	3.0 %	10.5 %
Hydropathy (GRAVY)	-0.044	-0.287	-0.215
Rosetta < -23 kcal/mol (%)	33 %	20 %	10 %
Amyloidogenic regions (Waltz)	1	2	0

Supplementary Table 3: Selection of other hydrogels, gelation time and elastic modulus.

Reference	Gel	Gelation time (h)	Elastic modulus (kPa)
Gao et al., 2018[16]	Gelatin	1	1.7
LeRoux et al., 1999[17]	Alginate (2 %)	1.5	2.31
Nayar et al., 2012[18]	Agar (0.5 %)	16	130
Valero et al., 2018[19]	Collagen (6 mg/ml)	3-4	0.254
Glassman et al., 2015[20]	Elastin-like polypeptide	15	1000

Supplementary Table 4: Amino acid sequences of proteins tested for hydrogel formation

Name	Amino acid sequence
His-NT2RepCT (Ref: [21])	MGHHHHHHMSHTTPWTNPGLAENFMNSFMQGLSSMPGFTASQLDDMSTIAQSMVQSI QSLAAQGRTSPNKLQALNMAFASSMAEIAASEEGGGSLSTKTSSIASAMSNAFLQTTGVVN QPFINEITQLVSMFAQAGMNDVSAGNSGRGQGGYGQGSGGNAAAAAAAAAAAAAAG QGGQGGYGRQSQGAGSAAAAAAAAAAAAAAGSGQGGYGGQGQGGYGQSGNSVTSGG YGYGTSAAAGAGVAAGSYAGAVNRLSSAEAASRVSSNIAAIASGGASALPSVISNIYSGVVAS GVSSNEALIQALLELLSALVHVLSSASIGNVSSVGVDSTLNVVQDSVGQYVG*
NT2RepCT	GSHMSHTTPWTNPGLAENFMNSFMQGLSSMPGFTASQLDDMSTIAQSMVQSIQSLAAQ GRTSPNKLQALNMAFASSMAEIAASEEGGGSLSTKTSSIASAMSNAFLQTTGVVNQPFINEIT QLVSMFAQAGMNDVSAGNSGRGQGGYGQGSGGNAAAAAAAAAAAAAAGQGGQGGY GRQSQGAGSAAAAAAAAAAAAAAGSGQGGYGGQGQGGYGQSGSVTSGGYGYGTSAAA GAGVAAGSYAGAVNRLSSAEAASRVSSNIAAIASGGASALPSVISNIYSGVVASGVSSNEALIQ ALLELLSALVHVLSSASIGNVSSVGVDSTLNVVQDSVGQYVG*
His-NTCT	MGHHHHHHMLVPRGSHTTPWTNPGLAENFMNSFMQGLSSMPGFTASQLDDMSTIAQS MVQSIQSLAAQGRTSPNKLQALNMAFASSMAEIAASEEGGGSLSTKTSSIASAMSNAFLQTT GVVNQPFINEITQLVSMFAQAGMNDVSAGNSVTSGGYGYGTSAAAGAGVAAGSYAGAVN RLSSAEAASRVSSNIAAIASGGASALPSVISNIYSGVVASGVSSNEALIQALLELLSALVHVLSSA SIGNVSSVGVDSTLNVVQDSVGQYVG*
NTCT	GSHTTPWTNPGLAENFMNSFMQGLSSMPGFTASQLDDMSTIAQSMVQSIQSLAAQGRTS PNKLQALNMAFASSMAEIAASEEGGGSLSTKTSSIASAMSNAFLQTTGVVNQPFINEITQLVS MFAQAGMNDVSAGNSVTSGGYGYGTSAAAGAGVAAGSYAGAVNRLSSAEAASRVSSNIA AIASGGASALPSVISNIYSGVVASGVSSNEALIQALLELLSALVHVLSSASIGNVSSVGVDSTLNV VQDSVGQYVG*
His-NT2Rep both from *E. australis* MaSp1	MGHHHHHHMSHTTPWTNPGLAENFMNSFMQGLSSMPGFTASQLDDMSTIAQSMVQSI QSLAAQGRTSPNKLQALNMAFASSMAEIAASEEGGGSLSTKTSSIASAMSNAFLQTTGVVN

	QPFINEITQLVSMFAQAGMNDVSAGNSGRGQGGYGQGSGGNAAAAAAAAAAAAAAAG QGGQGGYGRQSQGAGSAAAAAAAAAAAAAAGSGQGGYGGQGQGGYGQSGS*
His-2RepCT	MGHHHHHHMRNSGRGQGGYGQGSGGNAAAAAAAAAAAAAAAGQGGQGGYGRQSQG AGSAAAAAAAAAAAAAGSGQGGYGGQGQGGYGQSGSVTSGGYGYGTSAAAGAGVAA GSYAGAVNRLSSAEAASRVSSNIAAIASGGASALPSVISNIYSGVVASGVSSNEALIQALLELLS ALVHVLSSASIGNVSSVGVDSTLNVVQDSVGQYVG*
NT from *E. australis* MaSp1 (EMBL accession number AM259067)	GSGNSHTTPWTNPGLAENFMNSFMQGLSSMPGFTASQLDDMSTIAQSMVQSIQSLAAQG RTSPNKLQALNMAFASSMAEIAASEEGGGSLSTKTSSIASAMSNAFLQTTGVVNQPFINEITQ LVSMFAQAGMNDVS*
CT from *A. ventricosus* (GenBank accession number JX513956)	GSGNSTVAAYGGAGGVATSSSSATASGSRIVTSGGYGYGTSAAAGAGVAAGSYAGAVNRLS SAEAASRVSSNIAAIASGGASALPSVISNIYSGVVASGVSSNEALIQALLELLSALVHVLSSASIG NVSSVGVDSTLNVVQDSVGQYVG*
His-NT-ThrCleav-2Rep (Rep EMBL accession number AJ973155)	MGHHHHHHMSHTTPWTNPGLAENFMNSFMQGLSSMPGFTASQLDDMSTIAQSMVQSI QSLAAQGRTSPNKLQALNMAFASSMAEIAASEEGGGSLSTKTSSIASAMSNAFLQTTGVVN QPFINEITQLVSMFAQAGMNDVSAGNSLVPRGSNSGRGQGGYGQGSGGNAAAAAAAAA AAAAAAGQGGQGGYGRQSQGAGSAAAAAAAAAAAAAAGSGQGGYGGQGQGGYGQSG S*
NT* (Ref: [22])	GSGNSHTTPWTNPGLAENFMNSFMQGLSSMPGFTASQLDKMSTIAQSMVQSIQSLAAQG RTSPNDLQALNMAFASSMAEIAASEEGGGSLSTKTSSIASAMSNAFLQTTGVVNQPFINEIT QLVSMFAQAGMNDVS*
NT^{A72R} (Ref: [23])	GSGNSHTTPWTNPGLAENFMNSFMQGLSSMPGFTASQLDDMSTIAQSMVQSIQSLAAQG RTSPNKLQALNMRFASSMAEIAASEEGGGSLSTKTSSIASAMSNAFLQTTGVVNQPFINEITQ LVSMFAQAGMNDVS*
His-NT-L6 (Ref: [24])	MGHHHHHHMSHTTPWTNPGLAENFLNSFLQGLSSMPGFTASQLDDLSTIAQSLVQSIQSL AAQGRTSPNKLQALNMAFASSLAEIAASEEGGGSLSTKTSSIASALSNAFLQTTGVVNQPFIN EITQLVSMFAQAGMNDVSA*

NT^{FlSp} from *Trichonephila clavipes* GenBank accession number AWK58737.1	GSGNASQSPFSNPNTAEAFARSFVSNIVSSGEFGAQGAEKFDDIIQSLIQAQSMGKGRHDTK<br>ADAKAMQVALASSIAELVIAESSGGDVQRKTNVISNALRNALMSTTGSPNEEFVHEVQDLIQ<br>MLSQEQINEV*
NT^{MiSp} *A. ventricosus* (Ref: [25])	GSGNSQPIWTNPNAAMTMTNNLVQCASRSGVLTADQMDDMGMMADSVNSQMQKM<br>GPNPPQHRLRAMNTAMAAEVAEVVATSPPQSYSAVLNTIGACLRESMMQATGSVDNAFT<br>NEVMQLVKMLSADSANEVST*
His-NT-GFP	MGHHHHHHMSHTTPWTNPGLAENFMNSFMQGLSSMPGFTASQLDDMSTIAQSMVQSI<br>QSLAAQGRTSPNKLQALNMAFASSMAEIAASEEGGGSLSTKTSSIASAMSNAFLQTTGVVN<br>QPFINEITQLVSMFAQAGMNDVSAGNSKGEELFTGVVPILVELDGDVNGHKFSVSGEGEGD<br>ATYGKLTLKFICTTGKLPVPWPTLVTTLTYGVQCFSRYPDHMKQHDFFKSAMPEGYVQERTIF<br>FKDDGNYKTRAEVKFEGDTLVNRIELKGIDFKEDGNILGHKLEYNYNSHNVYIMADKQKNGIK<br>VNFKIRHNIEDGSVQLADHYQQNTPIGDGPVLLPDNHYLSTQSALSKDPNEKRDHMVLLEFV<br>TAAGITLGMDELYKLIN*
His-NT-PNP (GenBank accession number EFD1028701.1)	MGHHHHHHMSHTTPWTNPGLAENFMNSFMQGLSSMPGFTASQLDDMSTIAQSMVQSI<br>QSLAAQGRTSPNKLQALNMAFASSMAEIAASEEGGGSLSTKTSSIASAMSNAFLQTTGVVN<br>QPFINEITQLVSMFAQAGMNDVSAGNSGSGSGSGSGSGSGSGSMATPHINAEMGDFADV<br>VLMPGDPLRAKYIAETFLEDAREVNNVRGMLGFTGTYKGRKISVMGHGMGIPSCSIYTKELIT<br>DFGVKKIIRVGSCGAVLPHVKLRDVVIGMGACTDSKVNRIRFKDHDFAAIADFDMVRNAVD<br>AAKALGIDARVGNLFSADLFYSPDGEMFDVMEKYGILGVEMEAAGIYGVAAEFGAKALTICT<br>VSDHIRTHEQTTAAERQTTFNDMIKIALESVLLGDKEGSGSGSGSGSGSGSGS*

References

1. Kricheldor, H. R. & Müller, D. Secondary Structure of Peptides. 3. 13C NMR Cross Polarization/Magic Angle Spinning Spectroscopic Characterization of Solid Polypeptides. *Macromolecules* **16**, 615–623 (1983).
2. Wang, Y. Probability-based protein secondary structure identification using combined NMR chemical-shift data. *Protein Science* **11**, 852–861 (2002).

3. Goldschmidt, L., Teng, P. K., Riek, R. & Eisenberg, D. Identifying the amylome, proteins capable of forming amyloid-like fibrils. *Proc Natl Acad Sci U S A* **107**, 3487–3492 (2010).
4. Maurer-Stroh, S. *et al.* Exploring the sequence determinants of amyloid structure using position-specific scoring matrices. *Nature Methods* **7**, 237–242 (2010).
5. Rammensee, S., Huemmerich, D., Hermanson, K. D., Scheibel, T. & Bausch, A. R. Rheological characterization of hydrogels formed by recombinantly produced spider silk. *Applied Physics A: Materials Science and Processing* **82**, 261–264 (2006).
6. Schacht, K. & Scheibel, T. Controlled hydrogel formation of a recombinant spider silk protein. *Biomacromolecules* **12**, 2488–2495 (2011).
7. Schacht, K. *et al.* Biofabrication of cell-loaded 3D spider silk constructs. *Angew Chem Int Ed Engl* **54**, 2816–2820 (2015).
8. Qian, Z. G., Zhou, M. L., Song, W. W. & Xia, X. X. Dual Thermosensitive Hydrogels Assembled from the Conserved C-Terminal Domain of Spider Dragline Silk. *Biomacromolecules* **16**, 3704–3711 (2015).
9. DeSimone, E., Schacht, K. & Scheibel, T. Cations influence the cross-linking of hydrogels made of recombinant, polyanionic spider silk proteins. *Materials Letters* **183**, 101–104 (2016).
10. Thamm, C., DeSimone, E. & Scheibel, T. Characterization of Hydrogels Made of a Novel Spider Silk Protein eMaSp1s and Evaluation for 3D Printing. *Macromolecular Bioscience* **17**, (2017).
11. DeSimone, E., Schacht, K., Pellert, A. & Scheibel, T. Recombinant spider silk-based bioinks. *Biofabrication* **9**, 44104 (2017).
12. Humenik, M., Preiß, T., Gödrich, S., Papastavrou, G. & Scheibel, T. Functionalized DNA-spider silk nanohydrogels for controlled protein binding and release. *Materials Today Bio* **6**, (2020).
13. Song, W. W. *et al.* On-Demand Regulation of Dual Thermosensitive Protein Hydrogels. *ACS Macro Letters* **10**, 395–400 (2021).
14. Neubauer, V. J., Trossmann, V. T., Jacobi, S., Döbl, A. & Scheibel, T. Recombinant Spider Silk Gels Derived from Aqueous–Organic Solvents as Depots for Drugs. *Angewandte Chemie - International Edition* **60**, 11847–11851 (2021).
15. Wilkins, M. R. *et al.* Protein Identification and Analysis Tools in the ExPASy Server. in *2-D Proteome Analysis Protocols* 531–552 (Humana Press, 1967). doi:10.1385/1-59259-584-7:531.
16. Gao, T. *et al.* Optimization of gelatin-alginate composite bioink printability using rheological parameters: A systematic approach. *Biofabrication* **10**, (2018).
17. LeRoux, M. A., Guilak, F. & Setton, L. A. Compressive and shear properties of alginate gel: Effects of sodium ions and alginate concentration. *Journal of Biomedical Materials Research* **47**, 46–53 (1999).
18. Nayar, V. T., Weiland, J. D., Nelson, C. S. & Hodge, A. M. Elastic and viscoelastic characterization of agar. *Journal of the Mechanical Behavior of Biomedical Materials* **7**, 60–68 (2012).
19. Valero, C., Amaveda, H., Mora, M. & García-Aznar, J. M. Combined experimental and computational characterization of crosslinked collagen-based hydrogels. *PLoS ONE* **13**, 1–16 (2018).

20. Glassman, M. J. & Olsen, B. D. Arrested Phase Separation of Elastin-like Polypeptide Solutions Yields Stiff, Thermoresponsive Gels. *Biomacromolecules* **16**, 3762–3773 (2015).
21. Andersson, M. *et al.* Biomimetic spinning of artificial spider silk from a chimeric minispidroin. *Nature Chemical Biology* **13**, 262–264 (2017).
22. Kronqvist, N. *et al.* Efficient protein production inspired by how spiders make silk. *Nature Communications* **8**, 15504 (2017).
23. Jaudzems, K. *et al.* PH-dependent dimerization of spider silk N-terminal domain requires relocation of a wedged tryptophan side chain. *Journal of Molecular Biology* **422**, 477–487 (2012).
24. Heiby, J. C., Goretzki, B., Johnson, C. M., Hellmich, U. A. & Neuweiler, H. Methionine in a protein hydrophobic core drives tight interactions required for assembly of spider silk. *Nature Communications* **10**, 4378 (2019).
25. Chen, G. *et al.* Full-Length Minor Ampullate Spidroin Gene Sequence. *PLoS ONE* **7**, e52293 (2012).
