# Supplementary material for: Spidroin N-terminal domain forms amyloid-like fibril based hydrogels and provides a protein immobilization platform
Source: Nat Commun. 2022 Aug 15;13:4695. doi: 10.1038/s41467-022-32093-7 (PMC9378615; doi:10.1038/s41467-022-32093-7)
Supplement: Supplementary file 3 — Reporting Summary [file 41467_2022_32093_MOESM3_ESM.pdf]

nature portfolio

Corresponding author(s): Anna Rising

Last updated by author(s): 27/6/22

Reporting Summary

Nature Portfolio wishes to improve the reproducibility of the work that we publish. This form provides structure for consistency and transparency in reporting. For further information on Nature Portfolio policies, see our Editorial Policies and the Editorial Policy Checklist.

Statistics

For all statistical analyses, confirm that the following items are present in the figure legend, table legend, main text, or Methods section.

n/a	Confirmed	
☐	☒	The exact sample size (*n*) for each experimental group/condition, given as a discrete number and unit of measurement
☐	☒	A statement on whether measurements were taken from distinct samples or whether the same sample was measured repeatedly
☐	☒	The statistical test(s) used AND whether they are one- or two-sided<br>*Only common tests should be described solely by name; describe more complex techniques in the Methods section.*
☒	☐	A description of all covariates tested
☒	☐	A description of any assumptions or corrections, such as tests of normality and adjustment for multiple comparisons
☐	☒	A full description of the statistical parameters including central tendency (e.g. means) or other basic estimates (e.g. regression coefficient) AND variation (e.g. standard deviation) or associated estimates of uncertainty (e.g. confidence intervals)
☐	☒	For null hypothesis testing, the test statistic (e.g. *F*, *t*, *r*) with confidence intervals, effect sizes, degrees of freedom and *P* value noted<br>*Give P values as exact values whenever suitable.*
☒	☐	For Bayesian analysis, information on the choice of priors and Markov chain Monte Carlo settings
☒	☐	For hierarchical and complex designs, identification of the appropriate level for tests and full reporting of outcomes
☒	☐	Estimates of effect sizes (e.g. Cohen's *d*, Pearson's *r*), indicating how they were calculated

Our web collection on statistics for biologists contains articles on many of the points above.

Software and code

Policy information about availability of computer code

Data collection	Zipper DB, Bruker OPUS (version 5.5)
Data analysis	Prism 9 (Version 9.4.0), Bruker OPUS (version 5.5), Spectragryph (v1.2.15), ImageJ (version 1.53j)

For manuscripts utilizing custom algorithms or software that are central to the research but not yet described in published literature, software must be made available to editors and reviewers. We strongly encourage code deposition in a community repository (e.g. GitHub). See the Nature Portfolio guidelines for submitting code & software for further information.

Data

Policy information about availability of data

All manuscripts must include a data availability statement. This statement should provide the following information, where applicable:

- Accession codes, unique identifiers, or web links for publicly available datasets
- A description of any restrictions on data availability
- For clinical datasets or third party data, please ensure that the statement adheres to our policy

Source data are provided for Fig. 1c, Figure 2a-c, Fig. 3a, b, e-g, Fig. 4, Fig. 5 b, d, f, Fig. 6, Supplementary Fig. 3, Supplementary Fig. 5a, d, Supplementary Fig. 6 and Supplementary Fig. 8. The data generated in this study have been deposited in the Zenodo database 10.5281/zenodo.6683653. The NMR data generated in this study have been deposited in the BMRBig repository with entry ID bmrbig36 https://bmrbig.bmrb.io/released/bmrbig36. GFP and PNP structures were taken from the PDB (GFP 2B3Q, 10.2210/pdb2B3Q/pdb; PNP 4RJ2, 10.2210/pdb4RJ2/pdb).

Field-specific reporting

Please select the one below that is the best fit for your research. If you are not sure, read the appropriate sections before making your selection.

☒ Life sciences ☐ Behavioural & social sciences ☐ Ecological, evolutionary & environmental sciences

For a reference copy of the document with all sections, see nature.com/documents/nr-reporting-summary-flat.pdf

Life sciences study design

All studies must disclose on these points even when the disclosure is negative.

Sample size	No sample size calculation was performed. In biochemistry it standard to perform experiments in triplicates, thus this is what we chose to do as well.
Data exclusions	No data were excluded form the analyses.
Replication	All attempts at replication of gelation were successful (NT and His-NT2RepCT were expressed and purified several times and tested for gelation at least 20 times each at 100 mg/ml).Rheology, turbidity measurements, ThT assay, seeding assay, FTIR spectroscopy and GFP-fluorescence were conducted in triplicates and all attempts at replication were successful. Duplicates were measured for PNP activity assay but the assay was performed repeatedly. TEM, NMR and CD spectroscopy were conducted once as it is standard in the biochemistry field.
Randomization	This is not relevant for our study as samples were measured by instruments thus human bias is excluded. No selection were done by humans thus no selection bias can occur either. This is not commonly done in experimental biochemistry work.
Blinding	Not relevant to our study as knowing the origin of the samples cannot lead to a change of the final results and it is not common practice in the field.

Reporting for specific materials, systems and methods

We require information from authors about some types of materials, experimental systems and methods used in many studies. Here, indicate whether each material, system or method listed is relevant to your study. If you are not sure if a list item applies to your research, read the appropriate section before selecting a response.

Materials & experimental systems

n/a	Involved in the study	
☒	☐	Antibodies
☒	☐	Eukaryotic cell lines
☒	☐	Palaeontology and archaeology
☒	☐	Animals and other organisms
☒	☐	Human research participants
☒	☐	Clinical data
☒	☐	Dual use research of concern

Methods

n/a	Involved in the study	
☒	☐	ChIP-seq
☒	☐	Flow cytometry
☒	☐	MRI-based neuroimaging
